# Quantifying the prevalence of frailty in English hospitals

**DOI:** 10.1136/bmjopen-2015-008456

**Published:** 2015-10-21

**Authors:** J Soong, AJ Poots, S Scott, K Donald, T Woodcock, D Lovett, D Bell

**Affiliations:** 1 NIHR CLAHRC Northwest London, Imperial College London, London, UK; 2 Royal College of Physicians, London, UK; 3 Oliver Wyman, London, UK

**Keywords:** Frailty, Ageing, HES, Acute, England, Prevalence

## Abstract

**Objectives:**

Population ageing has been associated with an increase in comorbid chronic disease, functional dependence, disability and associated higher health care costs. Frailty Syndromes have been proposed as a way to define this group within older persons. We explore whether frailty syndromes are a reliable methodology to quantify clinically significant frailty within hospital settings, and measure trends and geospatial variation using English secondary care data set Hospital Episode Statistics (HES).

**Setting:**

National English Secondary Care Administrative Data HES.

**Participants:**

All 50 540 141 patient spells for patients over 65 years admitted to acute provider hospitals in England (January 2005—March 2013) within HES.

**Primary and secondary outcome measures:**

We explore the prevalence of Frailty Syndromes as coded by International Statistical Classification of Diseases, Injuries and Causes of Death (ICD-10) over time, and their geographic distribution across England. We examine national trends for admission spells, inpatient mortality and 30-day readmission.

**Results:**

A rising trend of admission spells was noted from January 2005 to March 2013(daily average admissions for month rising from over 2000 to over 4000). The overall prevalence of coded frailty is increasing (64 559 spells in January 2005 to 150 085 spells by Jan 2013). The majority of patients had a single frailty syndrome coded (10.2% vs total burden of 13.9%). Cognitive impairment and falls (including significant fracture) are the most common frailty syndromes coded within HES. Geographic variation in frailty burden was in keeping with known distribution of prevalence of the English elderly population and location of National Health Service (NHS) acute provider sites. Overtime, in-hospital mortality has decreased (>65 years) whereas readmission rates have increased (esp.>85 years).

**Conclusions:**

This study provides a novel methodology to reliably quantify clinically significant frailty. Applications include evaluation of health service improvement over time, risk stratification and optimisation of services.

**Strengths and limitations of this study**

- This study is the first to attempt to use frailty syndromes as an operational definition within an English secondary care data set.
- The methodology uses whole population routinely collected data, with robust trend analysis examining coding reliability.
- This study is a retrospective analysis reliant on the accuracy, reliability and retrospective nature of coding within Hospital Episode Statistics.

## Introduction

People are living longer. At present, it is estimated that 16.1% of the European population is over the age of 65 years (>65 years), and this number is expected to rise to 22% by 2031.^1^ In the developed world, the increase is greatest in those over 80 years, and this equates to approximately 3 million people in the UK.^2^ In health terms patients >65 years now constitute two-thirds of the general hospital population, account for 40% of all hospital bed days and 65% of National Health Service (NHS) spend.^3^ Recent analysis suggests population ageing contributes directly to the increase in emergency admissions to hospitals.^4^

Associated with this demographic shift there has been an increase in comorbid chronic disease, functional dependence, disability, poorer quality of life and higher health care costs.^5^ ^6^ Patients in this category are often considered frail. Currently, there is no universally agreed operational definition for frailty.^7^ Frailty has been described as a clinical phenotype or a biophysical syndrome of accumulated deficit (frailty index). Phenotypic models describe frailty as specific clinical syndrome encompassing a cluster of characteristics, namely unintentional weight loss, exhaustion, weakness, slowness and low physical activity.^8^ The frailty index is characterised by decreased resistance to stressors resulting from the accumulation of deficit across multiple physiological systems, culminating in an increased risk of adverse outcomes.^9^ ^10^ Methodologies to reliably identify the ‘frail’ at-risk cohort within secondary care, both at patient and population level, are a current research priority.^11–13^

In clinical practice the terms Geriatric Giants,^14^ Geriatric Syndromes^15^ ^16^ or Frailty Syndromes^17^ are often used to describe clinically vulnerable group within the elderly. They likely represent high order clinical manifestations of multifactorial processes resultant from the accumulation and interaction of deficits and environmental factors. They include cognitive impairment, falls, mobility problems, pressure ulcers and incontinence. These syndromes, more prevalent in the elderly, confer a higher risk of death,^8^ institutionalisation,^18^ disability and poor quality of life.^15^ They are arguably the consequences of frailty, or the manifestation of clinically significant frailty.^19^ Current National guidelines for the care of the older person in acute care recommend using frailty syndromes as a possible methodology to assess for frailty.^17^ ^20^

In this study, we measure the trends for all hospital admissions, in-hospital death and readmissions for those over 65 years. We describe Frailty Syndromes^17^ ^20^ as an operational definition within the English secondary care data set Hospital Episode Statistics (HES) in order to examine the frailty burden between 2005 and 2012. In addition we describe the geospatial variation of frailty in English secondary healthcare settings. We compare our results with the existing literature on frailty prevalence and discuss possible applications of this methodology.

## Methods

### Data sources

HES is a national administrative database containing patient-level records of all admissions to NHS hospitals in England.^21^ It has high levels of data completeness and rigorous data cleaning processes to ensure data quality. Each record in HES corresponds to a finished consultant episode, during which a patient is under the care of an individual consultant. These episodes were aggregated into hospital spells covering a patient's total length of stay in a hospital (ie, a hospital admission) using established methodology.^22^

HES contains 20 fields per record for diagnoses codes that are defined using the tenth revision of the International Statistical Classification of Diseases, Injuries and Causes of Death (ICD-10). The first of these is the primary diagnosis, with the rest available for coding of comorbidities or complications. HES does not contain present-on-admission flags. We reviewed HES for ICD-10 diagnostic codes that could be grouped for frailty syndromes (see online supplementary appendix 1) in all 20 fields. We included only inpatients at acute non specialist hospital trusts, with elective and non-elective admissions for those 65 years and over >65 years. We excluded hyper-specialist hospitals and mental health units as they have a very different case-mix and data quality.^23^ Thus, we defined frailty as the presence of at least one frailty syndrome and within the cohort of patients greater than 65 years old.

Annual trend profiles were created for the grouped ICD-10 diagnostic codes from January 2005 to March 2013 to determine coding reliability and shifts (see online supplementary appendix 2). The spells were aggregated both by age-band (65–74; 75–84; >85 years) and monthly. Monthly data are visualised as simple line plots in the first instance. Office of National Statistics (ONS) databases were queried for population size estimates or census data where available.

### Study population

All hospital admissions for >65 years to English acute trusts between January 2005 and March 2013 (N=50 540 141 patient spells) were available for analysis.

### Temporal analysis

To analyse the variation present in these time-series data, statistical process control is used to separate special cause variation (signal) from common cause variation, an inherent property of all systems. The XmR chart is used as it is a method that is not dependent on data distributions or underlying assumptions.^24^ When analysing count data, daily averages for months were calculated to correct for unequal ‘areas of opportunity’; for example, a count of February admissions will be lower by virtue of fewer days in February, and daily averages account for the difference in available days. For percentage data, such a correction is attained through division by the denominator—all spells and all spells with frailty. Adjustments for seasonal variation are made, and seasonalised reference lines are plotted, for more natural interpretation of the charts. In this work, a standard rule set for detection of signal is adopted, using Microsoft Excel to construct the charts.^24^

### Geospatial analysis

Geo-location is the identification of real-world geographic location of an object. Postcodes of provider sites were used to geo-locate sites, and map elements were derived from open source data provided by Office for National Statistics. Geo-locations aggregated to Primary Care Trust (PCT) level were attached to counts of frailty syndromes for patients >65 years admitted to NHS acute providers in 2012 as this is the applicable unit for these data. Choropleths are thematic maps that shade or colour areas to represent classified values of specific phenomena. ESRI ArcMap V.10.2 software was used to create a choropleth map. Annual trend profiles for inpatient mortality and non-elective readmission within 30 days were plotted. This temporal range of April 2006 to December 2012 was selected due to changes in structure of health geographies within England in 2006,^26^ and to allow a sufficient follow up period to more accurately reflect the clinical outcomes listed above.

## Results

**Figure 1:**
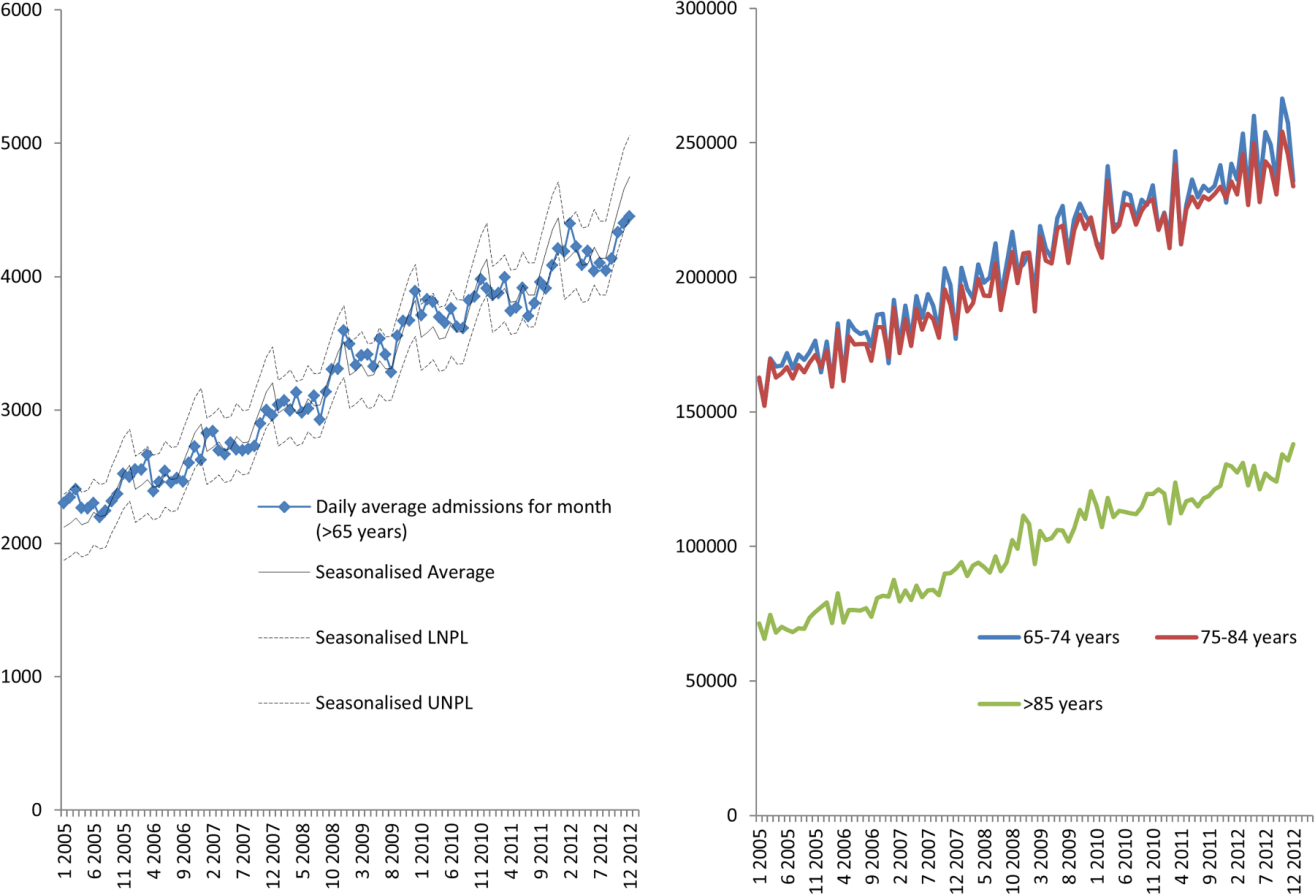
(A) Daily average admission spells for month and percentage total frailty burden for England NHS acute trusts. (B) The number and percentage of spells for patients >65 years by age-band admitted to English acute providers. LNPL, Lower Natural Process Limits; NHS, National Health Service; UNPL, Upper Natural Process Limits.

Between January 2005 and March 2013, there was a rising trend with daily average admissions for month increasing from over 2000 to over 4000 (figure 1A). There has been an increase in all age bands over this period, 65–74 increasing from 161 641 to 235 756, 75–84 increasing from 162 817 to 233 870 and >85 increasing from 71 396 to 137 991 (figure 1B). The relative proportion of total admissions has remained constant each age band at 40%, 40% and 20%, respectively. Examination of ONS data, (see online supplementary appendix 4) finds that in the general UK population the number of >64 years old in the population increased from 8 031 000 in 2005, to 905 179 in 2013. In 2005, the 65–74s represented 52% of those >65-year; in 2013 it was 54%; 75–84s were 36% and 33% 2005–2013; and >85s were 12% and 13%.

**Figure 2:**
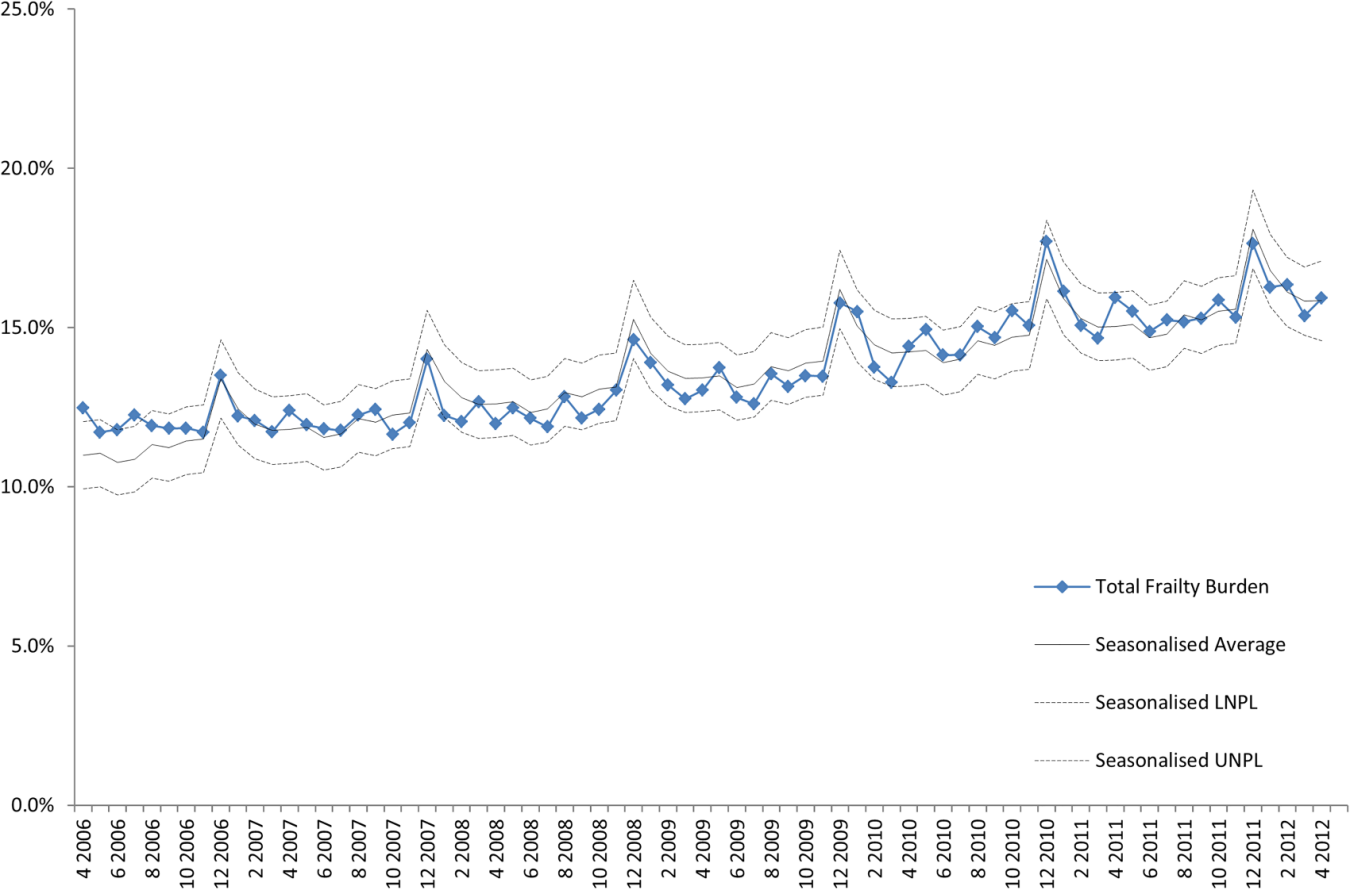
The percentage of admissions to English acute providers coded with at least one frailty syndrome. LNPL, Lower Natural Process Limits; UNPL, Upper Natural Process Limits.

Analysis of trends shows that the coded overall frailty burden, based on the coding of at least one frailty syndrome, has increased from 12% to 14% between January 2005 and March 2013. There is evidence of seasonal peaks during winter, partly explained by similar patterns in admission spells (figure 2).

**Figure 3:**
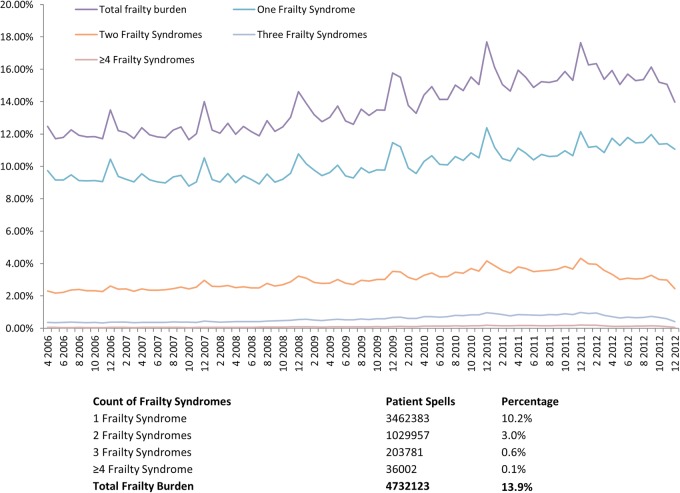
Trends for the prevalence of count of frailty syndromes and total frailty burden for patients >65 years admitted to NHS acute provider hospitals between April 2006 and December 2012. NHS, National Health Service.

**Figure 4:**
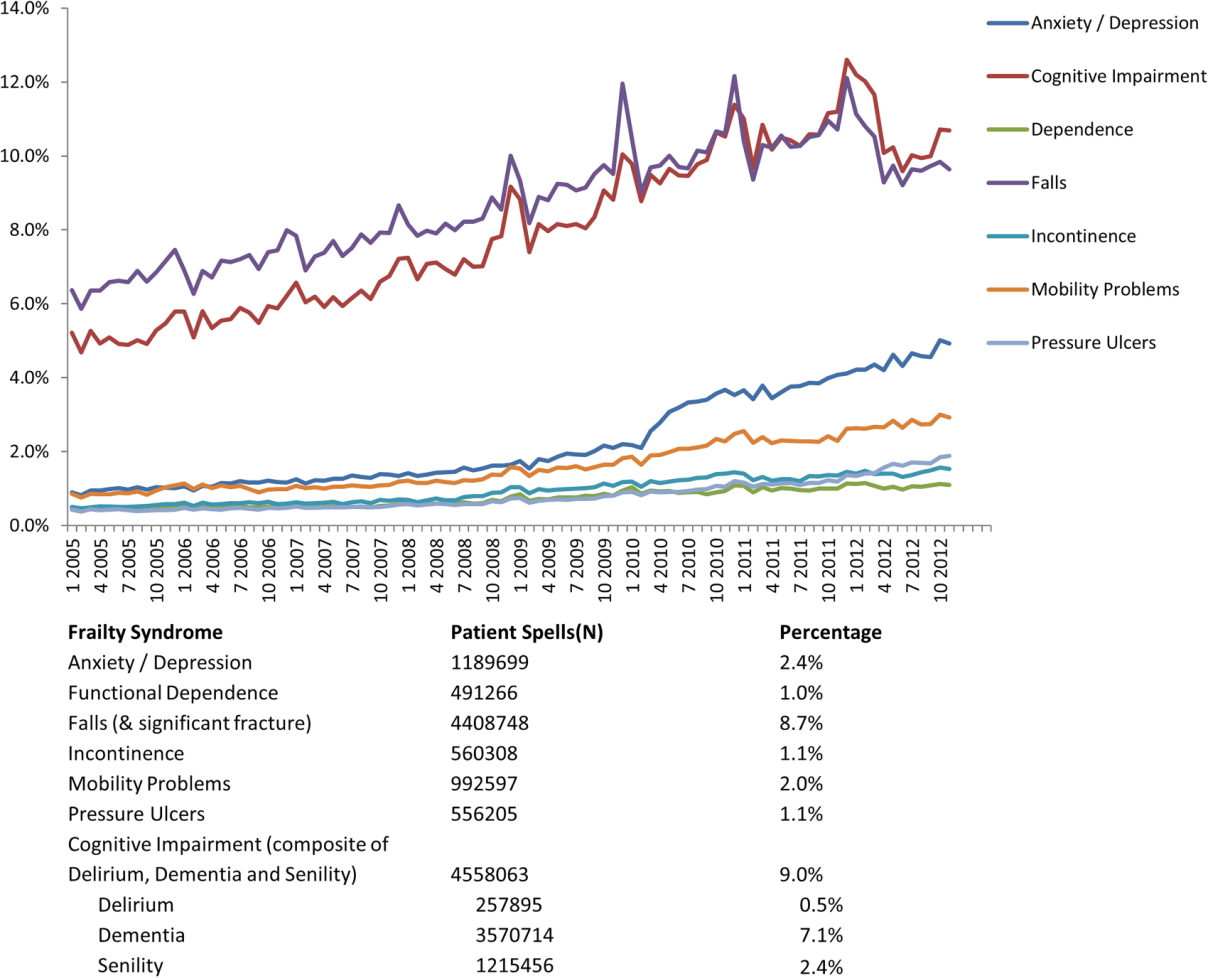
Trends for the prevalence of frailty syndromes for patients >65 years admitted to NHS acute provider hospitals between January 2005 and March 2013. NHS, National Health Service.

The coding of the frailty syndromes has increased between 2005 and 2013. Most patients had one frailty syndrome coded (figure 3) and the most common frailty syndromes described between 2005 and 2013 were cognitive impairment and falls (including significant fracture) with cognitive impairment increasing to the same levels as falls representing approximately 10% of all spells in the those >65 years. Anxiety and/or depression has increased particularly from 2010 (2.4%) to 2013 (>4%) (figure 4). There is a persistent and steady rise in coding for mobility problems.

Evaluating the frailty syndromes individually, the very elderly (>85 years) represent between 40% and 50% of the spells coded for that syndrome, with rising trend. The exception to this was anxiety and/or depression syndrome, which exhibited a rising trend in the 65–74 s, and the 75–84 s accounted for the largest group (see online supplementary appendix 3). Age-band stratification shows that cognitive impairment and falls in age-bands >85 years and 75–84 years account for a large majority of coded frailty syndromes within this cohort. These four groups accounted for 60.2% of frailty syndromes coded over this time period (N=7 399 671)

**Figure 5:**
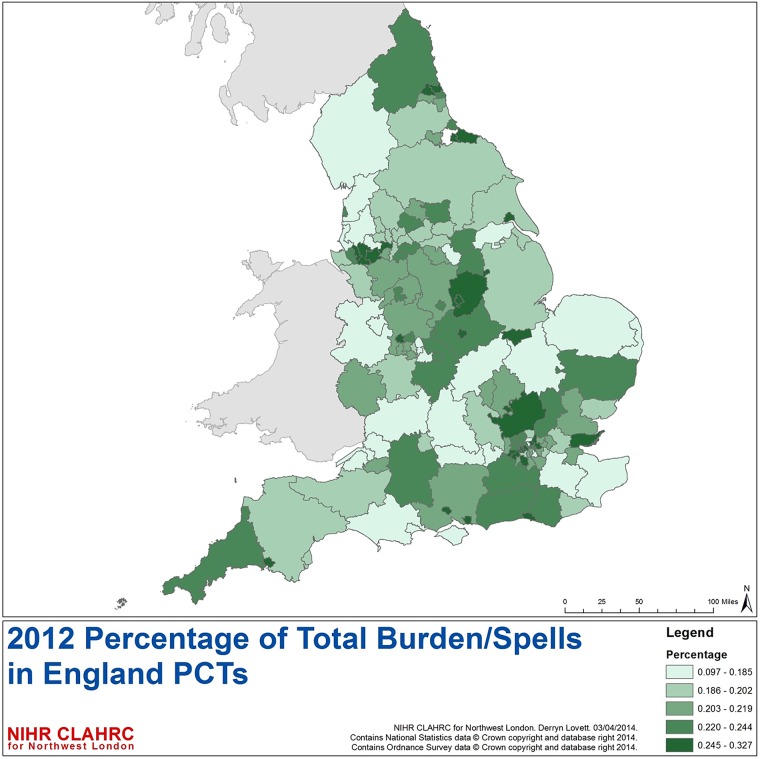
Percentage of spells for patients >65 years with admission to NHS acute trusts with at least one frailty syndrome by PCT by quintiles (numerator=admission spells with at least one frailty syndrome; denominator=total admission spells to NHS acute trusts within English PCT). NHS, National Health Service; PCT, Primary Care Trust.

Geographic variation in the frailty burden across admission spells in England was seen based on the 2012 HES data (figure 5). For patients >65-year admitted to England Acute providers, the highest levels of frailty are seen in the Northeast, Central and South Coast. The top five PCTs for highest admissions numbers are Nottingham City, Halton & St Helens, Warrington, Waltham Forrest and Wolverhampton city.

**Figure 6:**
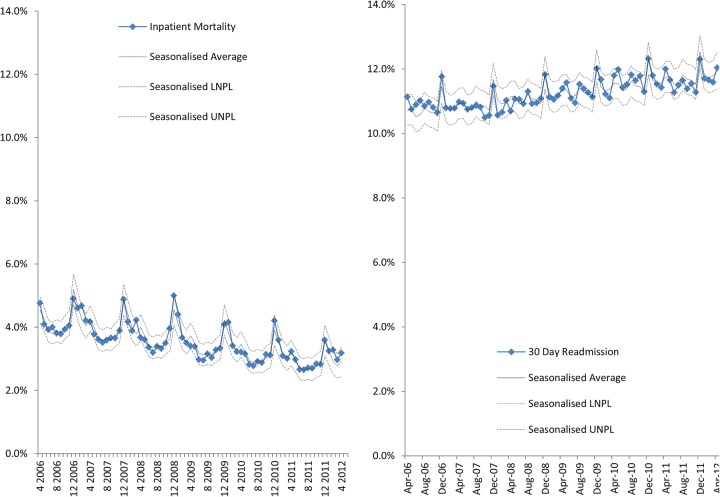
(A) Percentage of spells with inpatient mortality admitted to English providers and (B) non-elective 30-day readmission in patients >65 years admitted to English acute providers.

**Figure 7:**
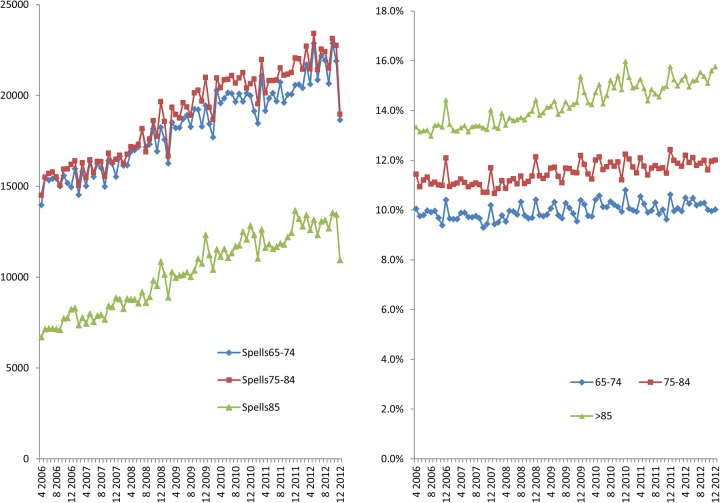
Number and percentage non-elective readmissions in patient >65 years admitted to NHS acute providers by age-band. NHS, National Health Service.

Between April 2006 to December 2012, 1 160 299 (3.4%) spells were associated with inpatient mortality, though a decreasing trend is observed for example, April 2006 (N=15 042) to April 2012(N=14 437) (figure 6A). Non-elective re-admission rates within 30 days of discharge have increased for all admissions > 65 years from approximately 11–12% (figure 6B). The rates of readmission increased across the age bands >65 years (10%), 75–84 (12%) and >85 (14%). Though the overall number of very elderly (>85 years) with non-elective 30-day readmission is lower than the other two age-bands, they have more readmissions (figure 7).

## Discussion

Frailty is often defined as a clinical state in which there is an increase in an individual’s vulnerability for adverse events and harm when exposed to a stressor.^25^ It is distinct but related to disability and comorbidity.^26^ ^27^ Some approaches to the measurement of frailty have been characteristically biophysical with emphasis on detection of the consequences of sarcopaenia and chronic inflammation-malnutrition.^8^ Another approach is to measure frailty in relation to the clinical consequences of accumulated loss and insufficiency in ageing individuals(ie, the relationship to mortality and adverse outcomes).^28^ Both approaches appear complementary^29^ and overlap, though not completely.^30^ Frailty measurement is problematic in the acute care setting. High levels of disease acuity on top of chronic multimorbidity, multidimensional complexity and diagnostic uncertainty are challenging for healthcare systems, with increasing evidence and concern for compromised patient safety, quality of care and experience.^31–34^

We have examined the prevalence of frailty syndromes within English HES data from both a temporal and geospatial point of view. Temporal analysis, it allows us to observe shifts in diagnostic coding, and observe trend in signal changes over time. Spatial analysis allows us to explore geographic heterogeneity of frailty syndrome prevalence, with consequent implications for service provision and equity of care.

Comparison with ONS data, the corresponding admissions to English acute providers for patients with frailty syndromes is larger than might be expected by demographic shift associated with ageing. Additionally, 75–84 s make up approximately one-third of the population of those over 65 years, but have 40% of the admissions, and >85s are approximately 13% of the population of those over 65 years but have 20% of the admissions.

This study has focused on patients admitted to hospital >65 years in England to better understand the impact of frailty syndromes. To the authors knowledge, this is the first study to examine the prevalence of frailty syndromes for patients >65 years across England. This study confirms increasing number of >65 years admitted to hospital (elective and non-elective). The relative burden of coded frailty syndromes has increased over this period with cognitive impairment increasing to similar levels to falls. Anxiety and/or depression is also increasing in this group.

When complex systems fail (biological or otherwise), high-order functions can be first disrupted.^35^ Frailty syndromes represent the clinical manifestation of high-order disruption, providing a useful clinical marker of multidimensional deficit accumulation. The overall prevalence rate of frailty syndromes found in this study is 13.9%. Between 2005 and 2013, though there has been an increase in the numbers of patients admitted >65 years, the percentage by age band has remained stable, thus not suggesting major drift towards older age groups within the older population. However, within the >65 years group, frailty syndromes are more prevalent with the older age bands.

Prevalence rates of frailty vary depending on population and operational definition used in reported studies. Reported prevalence in community dwelling adults varies tremendously (from 4.0% to 59.1%).^36^ A recent systematic review reported pooled frailty prevalence across *21 community dwelling study cohorts* as 10.7% (N=61 500).^36^ The recent Survey of Health, Ageing and Retirement in Europe (SHARE) study reported frailty prevalence as 4.1% in *community dwelling adults >50 years*(N=16 584) in 10 European countries (prevalence of 17% in those over 65 years).^37^ In the UK, the Hertfordshire Cohort Study^38^ reported an overall prevalence of 6.3% for 638 *community dwelling 64–74-year-olds*, while the English Longitudinal study of ageing^39^ reported a prevalence of 8% and 13% for 3055 *community dwelling over 65-year-olds* (using the Phenotype^8^ and Frailty Index^10^ definitions, respectively).

The prevalence of inpatient frailty in our study was lower than expected from smaller reported clinical studies within secondary care (range 24.7%—80%): (n=220 *>70 years admitted to acute geriatric ward from Emergency department,*^40^ (n=6701)40% (Phenotype) and 32.5%(SOF;^41^ (n=1388 *>70 years admitted to cardiology service,*^42^ (n=900 827 % (Phenotype) and 63% (Frailty Scale^43^); (n=298 *>75 years admitted to five different specialist wards*, 50–80% (Groningen Frailty Index^44^ ^45^); *(n=307 >75 years with diagnosed non-ST elevation myocardial infarction,^46^* 48.5% (n=2305 >65 years Clinical Frailty Scale^47^); (n=752 *medical inpatients >75 years.^48^* In the UK, two recent studies^12^ ^13^ reported frailty prevalence for n=667 *patients >70 years admitted to Acute Medical Units(AMU*) at 69% (ISAR,^49^ 17.9% (Phenotype), 66.4% (SOF), 24.9% (Avila-Funes), 24.1% (Rothman) and 30.9% (Frailty index). Importantly, these studies mainly consisted of non-elective admissions, while our study cohort comprised of elective and non-elective admissions to hospital. However, it may be that this methodology truly underestimates the prevalence of frailty within HES.

Not all frailty syndromes are observed, within HES, to be equally prevalent, nor do they appear to be increasing at the same rate. The observed differences and increase in frailty syndromes in this study (figure 4) may reflect improvements in coding practice within HES due to the introduction of Healthcare Resource Group (HRG V.4 introduced in April 2007) and Payment by Results (since April 2009). The national dementia strategy was also published in 2009. However, this observed rising trend may also reflect a genuine increase in number of diagnosis. Correlation with clinical data sets for comparison is consequently a necessary research priority.

The frailty syndromes are more prevalent in the very elderly (>85), with a rising trend. The exception to this is anxiety and/or depression, where the most prevalent age-band is 75–84 years, which exhibits a declining trend, while the increase in this anxiety and/or depression from 2010 appears to mainly be in the 65–74 age-band, a pattern noted independently by the HSCIC.^50^ Correlation with clinical data sets is warranted to ensure accuracy.

This analysis suggests that coexistence of multiple frailty syndromes is uncommonly coded within HES; even though we used coded frailty syndromes within all 20 of HES diagnostic domains, incomplete coding may still be a cause, as not all morbidities will be acknowledged and coded for each admission, only those deemed relevant to care at that time. However, it has been noted that accumulation of deficit beyond a certain level is incompatible with survival,^51^ and thus multimorbidity would have a ceiling effect. Further investigation on multiple frailty syndromes could be profitable.

Inpatient mortality trends in this population exhibit seasonality with peaks during winter, which persist after adjustment for number of admissions (spells). These peaks, coupled with rising 30-day readmissions (particularly in the very elderly) suggest differences in service provision over the year. A question arises here: is this seasonality appropriate for the UK population and the provision of care?

Geographic variation in frailty burden appears to be in keeping with known distribution of prevalence of the English elderly population and location of NHS acute provider sites, particularly within urban areas. Healthcare providers and commissioners should consider their local populations when planning services, where frailty may be a larger consideration than other locations. Further study into environmental factors in relation to frailty is a necessary next step.

### Limitations

This study is a retrospective analysis reliant on data coded from hospital data warehouses, and subsequently cleaned into HES. As such, its validity is dependent on accuracy of data coding. Including all 20 diagnostic coding fields may help to mitigate this, but correlation with clinical data sets may be warranted for local investigations. Resultant prevalence rates described may underestimate frailty syndromes in this population.

Anxiety and/or depression was only recently recognised as a geriatric syndrome by the Education Committee Writing Group of the American Geriatrics Society.^16^ It appears to fulfil several criteria that makes it an attractive putative candidate for a frailty syndrome:^51^ poor mental health is often associated with chronic physical deficits,^52^ it appears to increase with age (figure 4), it is associated with adverse outcome,^53^ it is neither to rare or too common (figure 4) Recent study has linked it to frailty^52^ ^54^ in older persons, though comprehensive study of its relationship to adverse outcomes with relation to frailty is still lacking. Further study, including correlation with clinical data sets, is warranted.

### Conclusion

To our knowledge this study is the first to attempt to use frailty syndromes as an operational definition within an English secondary care data set. While the study is dependent on the accuracy, reliability and retrospective nature of coding within HES, its strengths include being a whole population analysis, with robust trend analysis examining coding reliability. It utilises routinely collected data and is comprehensive in its coding of frailty within all of the diagnostic coding positions in the HES data set. Future studies to correlate with clinical data sets are needed to further investigate the phenomena discovered in this study.

This study provides a methodology to reliably quantify frailty. Applications include the ability to evaluate the effect of interventions over time allowing for health service quality improvement. Geographic analysis allows providers and payers to highlight areas of need, unmet or otherwise for more intelligent targeting of resources, from a public health or clinical perspective. A reliable and quantifiable metric for frailty enables the development of risk-prediction models and clinical scoring systems that will aid targeted interventions to vulnerable populations that will benefit most.
